# Zoonotic *Ancylostoma ceylanicum* Infection in Coyotes from Guanacaste Conservation Area, Costa Rica, 2021

**DOI:** 10.3201/eid3006.231618

**Published:** 2024-06

**Authors:** Patsy A. Zendejas-Heredia, Joby Robleto-Quesada, Alberto Solano, Alicia Rojas, Vito Colella

**Affiliations:** University of Melbourne, Parkville, Victoria, Australia (P.A. Zendejas-Heredia, V. Colella);; Centro de Investigación en Enfermedades Tropicales, University of Costa Rica, San José, Costa Rica (J. Robleto Quesada, A. Solano, A. Rojas);; Universidad de Costa Rica, Montes de Oca, San José (A. Solano, A. Rojas).

**Keywords:** hookworms, zoonoses, parasites, enteric infections, coyotes, nematodes, disease prevention, Costa Rica, Ancylostoma ceylanicum

## Abstract

*Ancylostoma ceylanicum* is the second most common hookworm infecting humans in the Asia-Pacific region. Recent reports suggest presence of the parasite in the Americas. We report *A. ceylanicum* infections in coyotes from the Guanacaste Conservation Area, Costa Rica. Our findings call for active surveillance in humans and animals.

The zoonotic hookworm *Ancylostoma ceylanicum* infects canids, felids, and humans in tropical regions of the world (*1*,*2*). Heavy infections may cause iron-deficiency anemia and bloody diarrhea as a result of ≈0.03 mL/worm/day blood loss caused by repeated blood meals taken from the intestines of infected persons by the parasites (*2*,*3*). Across the Asia-Pacific region, domestic and wild animals and humans, especially children and women of childbearing age, are at high risk for illness from infection with the parasite (*2*). The pooled proportion of *A. ceylanicum* hookworm–infected persons within the Asia Pacific is 12%, making it the second most common hookworm infecting humans in that region (*4*). However, the extent of its geographic distribution in the Americas and the range of animals this hookworm can infect are unknown. 

In studies dating to 1922, adult hookworms, later identified as *A. ceylanicum*, were recovered from human cadavers from Brazil and, in 1976, from animals necropsied in Suriname, suggesting that the parasite might have been present in the Americas more than a century ago (*2*). However, except for those cases, research on *A. ceylanicum* hookworms in the Americas has remained neglected for a long time; that prolonged absence in research might be attributable to use of traditional microscopy techniques for hookworm diagnosis in the region (*2*). Those techniques are unable to differentiate hookworms at the species level, thereby limiting more detailed information on their distribution. More recently, applying molecular techniques has increased identification of regions endemic for *A. ceylanicum* hookworms (*2*). For example, in 1 recent study, samples from patients in Ecuador initially screened for hookworms in 2000 were reexamined, and *A. ceylanicum* hookworms were molecularly identified (*5*). In addition, *A. ceylanicum* hookworms have been reported in France in a migrant from Colombia and a traveler returning from French Guiana (*6*); in Germany, the parasite was reported in a child from Colombia (*7*). Furthermore, in 2022, endemicity of *A. ceylanicum* hookworms was molecularly confirmed in dogs in Grenada, West Indies (*8*). Despite the discovery of *A. ceylanicum* hookworms in wildlife elsewhere, initially in a civet cat native to Sri Lanka and subsequently in Asia in golden cats and leopards and in dingoes in Australia (*9*), no studies have reported infections in wildlife in the Americas. 

## The Study

This project was approved by the National System of Conserved Areas of Costa Rica (R-SINAC-ACG-003-2021). Costa Rica is in Central America within the equatorial tropical region. Among its 5 million residents, 766,000 are school-age children. Coyotes (*Canis latrans*) live around national parks and are widely distributed across the plains, mountains, forests, and tropics. To investigate the prevalence of hookworm and threadworm infections among coyotes, during May–October 2021, we collected and tested coyote scat from the Central Conservation Area in central and the Guanacaste Conservation Area in northwestern Costa Rica (*10*). To confirm coyotes were the origin of the collected fecal samples, we cross-referenced data from a separate study conducted on the same samples that employed restriction fragment-length polymorphism–based methods and metabarcoding analyses of the vertebrate 12S ribosomal RNA gene (*11*). 

We extracted genomic DNA from fecal samples (n = 111) using the QIAamp PowerFecal Pro DNA Kit (QIAGEN, https://www.qiagen.com) according to manufacturer instructions and added an extra initial bead-beating step. We performed 2 TaqMan (Thermo Fisher Scientific; https://www.thermofisher.com) probe–based real-time quantitative PCRs in duplicate to detect all present canine hookworm species (*A. ceylanicum*, *A. caninum*, *A. braziliense*, and *Uncinaria stenocephala*) and *Strongyloides* spp. threadworms, as described elsewhere (*10*,*12*). We subjected samples positive for *A. ceylanicum* to molecular characterization of a partial region of the *cox*1 gene (*13*). We downloaded nucleotide *cox*1 sequences from dogs, humans, and cats from GenBank to assess the phylogenetic relationship among *Ancylostoma* spp. parasites and hosts.

We used MEGA11 (https://www.megasoftware.net) for aligning sequences and determining the best-fit substitution model. We conducted phylogenetic analyses using MrBayes version 3.2.7a (https://nbisweden.github.io/MrBayes/download.html) for Bayesian and RAxML version 8.2.12 (https://github.com/stamatak/standard-RAxML) for maximum-likelihood inference. We annotated the resulting phylogenic tree using TreeViewer version 1.17.6 (https://treeviewer.org) and constructed a haplotype network using PopArt (https://github.com/jessicawleigh/popart). 

Of the 111 samples collected, 103 had sufficient feces for successfully extracting DNA, determined by amplifying a housekeeping gene of canids as DNA extraction control. Of those 103 samples, 59 (57.3%, 95% CI 46.6%–62.2%) harbored >1 zoonotic hookworms; *A. caninum* accounted for most infections (53.4%; 95% CI 43.3%–63.3%), followed by *U. stenocephala* (4.7%, 95% CI 1.5%–10.7%), and *A. ceylanicum* (2.8%, 95% CI 0.56%–8.05%). *Strongyloides* spp. threadworms were found in 3.8% (95% CI 1.04%–9.3%) of coyotes. 

**Figure 1:**
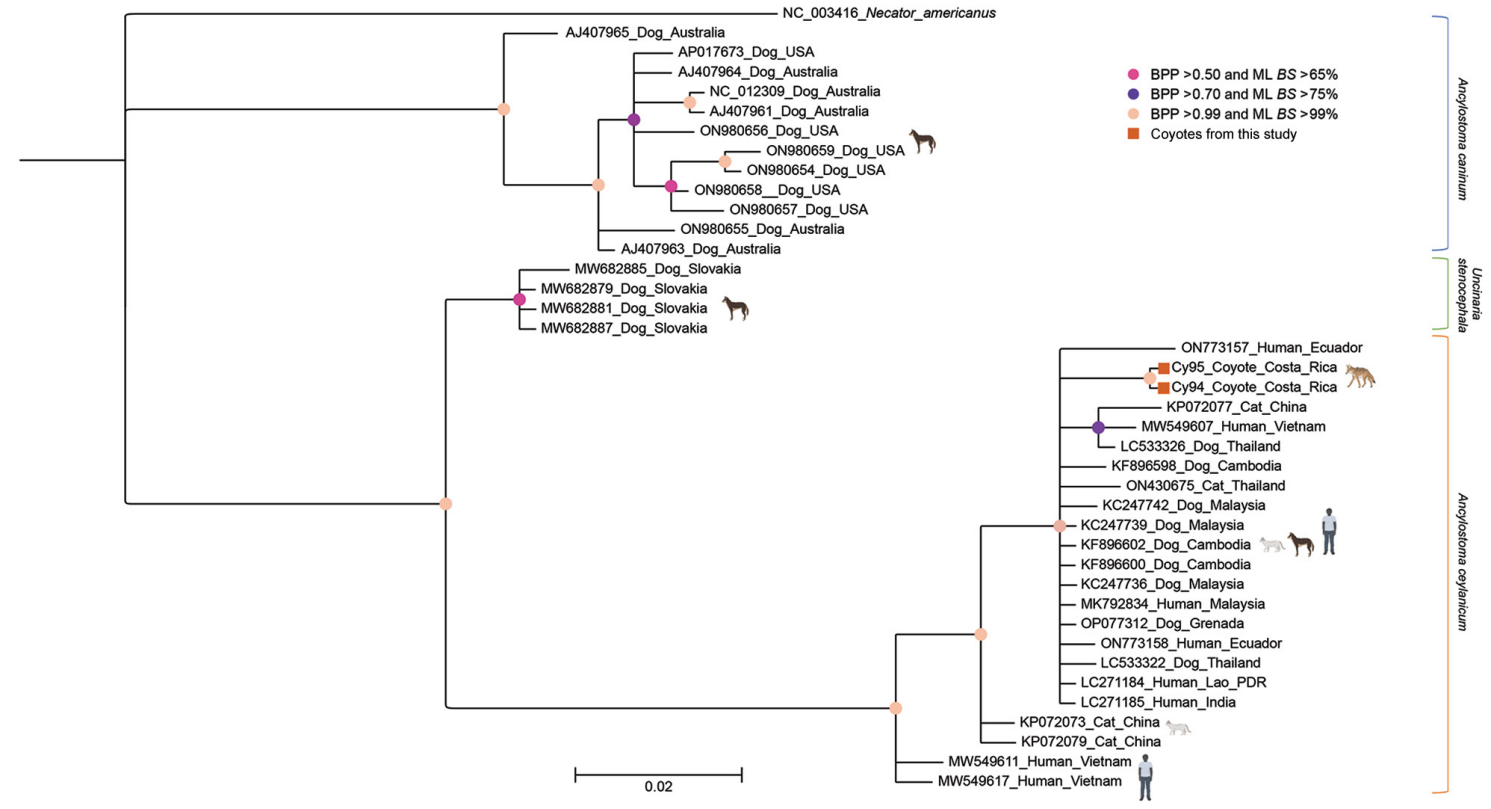
Phylogeny of *Ancylostoma ceylanicum* hookworms from coyotes in from Guanacaste Conservation Area, Costa Rica, 2021, and reference sequences. Gene tree shows a partial region of the mitochondrial *cox*1 gene for the hookworms *A. caninum*, *A. ceylanicum*, and *Uncinaria stenocephala*. Phylogenetic analyses were conducted using Bayesian inference in MrBayes v3.2.7a, employing the Hasegawa-Kishino-Yano substitution model with 4 gamma-distributed categories. Four independent Markov chains ran for 1,000,000 Markov chain Monte Carlo generations, with tree sampling occurring every 200 generations. The initial 25% of generated trees was considered burn-in, while the remaining trees were used to derive consensus trees. The analysis proceeded until the potential scale reduction factor approached 1, and the average SD of split frequencies was <0.01. For maximum-likelihood analysis, we used the rapid bootstrapping option with 10,000 iterations based on the Akaike information criterion. To root the tree, we included the human hookworm *Necator americanus* (GenBank NC_003416). Circles on nodes indicate BPP and ML *BS* percentages. Taxon names are annotated with GenBank accession numbers, host, and country of origin. Scale bar indicates the mean number of nucleotide substitutions per site. BPP, Bayesian posterior probability; ML *BS*, maximum-likelihood bootstrap support.

**Figure 2:**
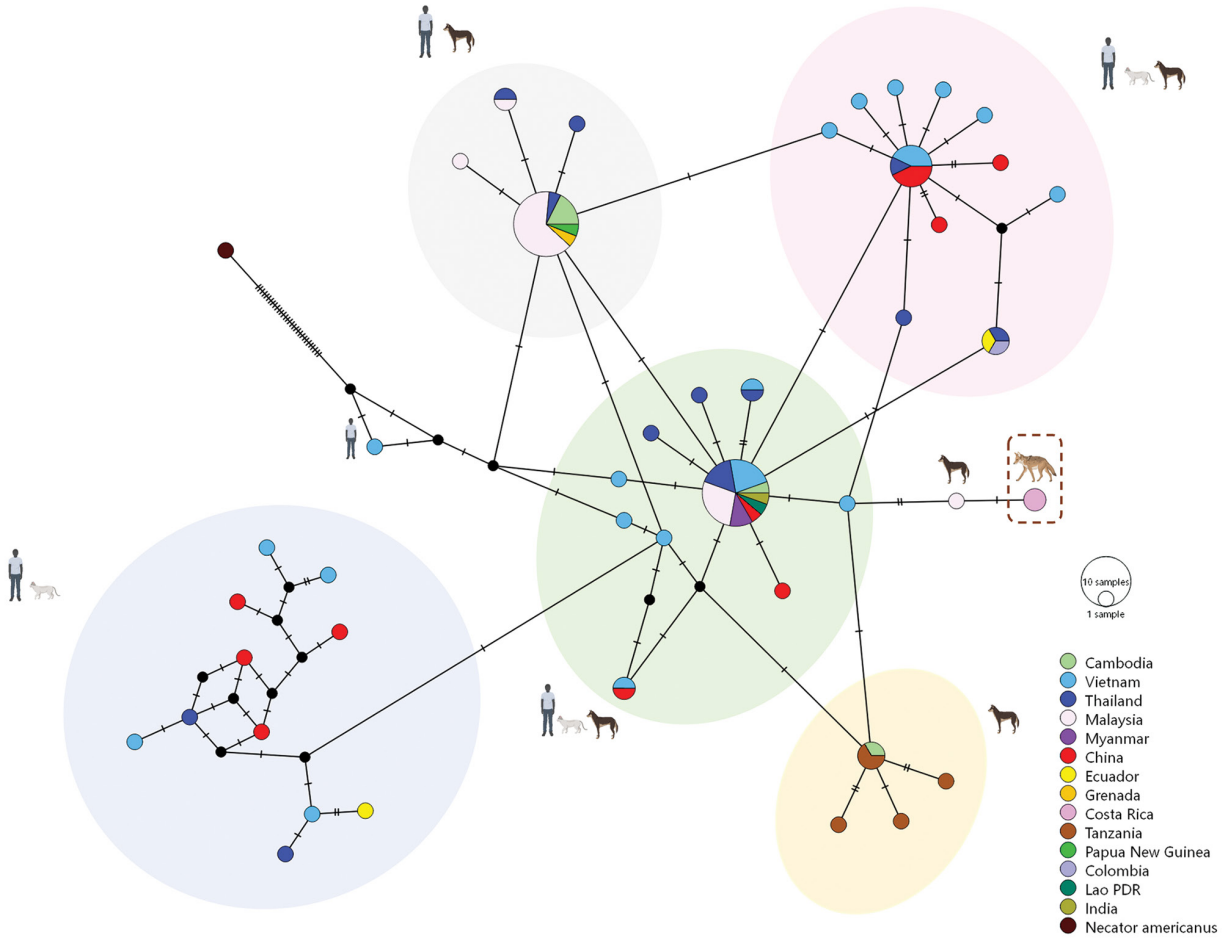
Median joining haplotype network showing global *Ancylostoma ceylanicum* sequences for cats, humans, and dogs and coyotes from Guanacaste Conservation Area, Costa Rica, 2021 (dashed box). Each circle represents a haplotype; circle size is proportional to the number of animals and persons with that haplotype, with the proportion found in each country indicated by color. A hatch mark across a line represents a mutation step between haplotypes. The legend illustrates color-coded haplotypes corresponding to each country. In addition, icons representing humans, dogs, cats, and coyotes are juxtaposed with each haplotype cluster to indicate host origins.

We obtained a 289-bp amplicon showing 99.6%–100% nucleotide identity with *A. ceylanicum* sequences from 2 positive *A. ceylanicum* samples (GenBank accession numbers OR801542 and OR801543). We determined phylogeny from 40 sequences that included *A. ceylanicum* sequences from dogs, cats, and humans, and *U. stenocephala* and *A. caninum* sequences from dogs. Phylogenetic analyses enabled separation of *A. ceylanicum* nucleotide sequences from coyote samples from Costa Rica from *A. caninum* and *U. stenocephala* sequences (Figure 1). Upon examining the median joining network of global *A. ceylanicum* sequences, we identified 5 distinct clusters, 4 of which were shared between domestic animals and humans and only 1 with dogs. We observed no clear population structure among countries represented by an admixture of haplotypes. *A. ceylanicum* haplotype from coyotes was unique but separated by only a few mutational steps from those from dogs and humans, and from zoonotic clusters (Figure 2). 

## Conclusions

We report zoonotic hookworm infections in coyotes from Costa Rica. Our data provide further evidence of the endemicity and local transmission of *A. ceylanicum* hookworm in the Americas and show that coyotes might play a role in transmitting this parasite to other animals. Even though coyotes are widely distributed across North and Central America and are established reservoirs of other zoonotic hookworms, such as *A. caninum* and *U. stenocephala* (*14*), their role as hosts of *A. ceylanicum* hookworm remains unexplored. Furthermore, detection of *A. ceylanicum* hookworms in wildlife poses questions about the parasite’s possible role as a disease agent in endangered animals, such as jaguars, giant anteaters, and Baird’s tapir, that live in sympatry in the Guanacaste Conservation Area (*15*). 

Costa Rica has effectively controlled soil-transmitted helminths in humans, and residents no longer require preventive chemotherapy. However, the Pan American Health Organization still recommends continuous monitoring and evaluation to maintain control of soil-transmitted helminths (*15*). Although eliminating the need for preventive chemotherapy constituted a great milestone, doing so leaves untreated persons vulnerable to infection with zoonotic helminths, including *A. ceylanicum*. We therefore advocate for increased surveillance of *A. ceylanicum* hookworm in domestic animals, wildlife, and humans in the Americas to monitor and prevent transmission of zoonotic hookworms. Expanded surveillance is crucial considering active transmission of *A. ceylanicum* hookworm in humans has been reported in Ecuador, another country listed by the Pan American Health Organization as no longer requiring preventive chemotherapy (*5*,*15*). In-depth population genetics studies are needed to assess whether increases in the reports of *A. ceylanicum* hookworms in the Americas are attributable to recent human and animal migration from areas where the parasite is highly endemic. More robust epidemiologic data on *A. ceylanicum* infection in domestic animals and humans would help public health agencies assess the extent of local transmission in Costa Rica and other regions of the Americas and the role of animals in transmitting the parasite to humans. 
